# Wnt11 plays an important role in the osteogenesis of human mesenchymal stem cells in a PHA/FN/ALG composite scaffold: possible treatment for infected bone defect

**DOI:** 10.1186/s13287-016-0277-4

**Published:** 2016-01-27

**Authors:** Hai Wang, Xiao-Qing He, Tao Jin, Yang Li, Xin-Yu Fan, Yi Wang, Yong-Qing Xu

**Affiliations:** The Third Military Medical University, Chongqing, 400038 China; Department of Orthopaedics, Kunming General Hospital of Chengdu Military Command, 650032 Kunming, China; Institute of Traumatology and Orthopaedics of PLA, 650032 Kunming, China

**Keywords:** Wnt11, infected bone defect, osteogenesis, alginate, porous hydroxyapatite, fibronectin

## Abstract

**Background:**

Infected bone defect poses a great challenge for orthopedists because it is difficult to cure. Tissue-engineered bone based on the human mesenchymal stem cells (hMSCs), has currently taken a promising treatment protocol in clinical practice. In a previous study, a porous hydroxyapatite/fibronectin/alginate (PHA/FN/ALG) composite scaffold displayed favorable biological properties as a novel scaffold, which was considered better than single-material scaffolds. In addition, Wnt11 has been demonstrated to play an important role in the development of osteoblasts, but until recently, its role in the osteogenic differentiation of hMSCs in infectious environment remained unclear.

**Methods:**

In this study, we constructed a PHA/FN/ALG composite scaffold with layer-by-layer technology. Furthermore, we also constructed Wnt11-silenced (RNAi) and -overexpressing hMSCs by lentiviral transduction. The gene transduction efficacy was confirmed by quantitative PCR assay and Western blot analysis. Tissue-engineered bone was constructed with hMSCs and PHA/FN/ALG composite scaffolds, and then was implanted into an infected bone defect model for evaluating the osteogenic capacity by quantitative PCR, gross observation, micro-CT and histology analysis.

**Results:**

All those cells showed similar adhesion abilities and proliferation capacities in scaffolds. After tissue-engineered bone implantation, there were high levels of systemic inflammatory factors in vivo, which significantly declined three days after antibiotic therapy. One or two months after implantation, the results of osteogenic-related gene analyses, gross observation, micro-CT and histology consistently showed that the Wnt11 over-expression hMSC group displayed the strongest osteogenesis capacity, whereas the Wnt11-RNAi hMSC group displayed inferior osteogenesis capacity, when compared with the other cell-containing groups. However, the blank control group and the only composite scaffold without cell implantation group both showed extremely weak osteogenesis capacity.

**Conclusion:**

Our results revealed that the Wnt11 gene plays an important role in hMSCs for enhancing the osteogenesis in an infectious environment.

## Background

Infected bone defect remains one of the greatest challenges for orthopedists because it is clinically difficult to treat and eradicate [1]. It has been reported that approximately 5–10 % of open fractures will evolve into osteomyelitis or bone nonunion, which often subsequently lead to infected bone defects [2, 3]. Because of the long treatment time and low cure rate, infected bone defects can be devastating and associated with physical and psychological damage for patients as well as economic loss [4].

The common treatment modalities for infected bone defect mainly include repeated debridement, which is the one of the most important surgical principles [5]. Meanwhile, traditional antibiotic therapy has been employed generally, although it is difficult to produce an effective local antibiotic concentration despite the high serum concentration [5, 6]. Therefore, when necessary, local antibiotic delivery systems such as the antibiotic polymethyl methacrylate (PMMA) bead, etc., are often used to promote the local antibiotic concentration for killing bacteria, and create a certain therapeutic effect [5]. However, the therapeutic efficacy is still not very satisfactory. Therefore, it is important to further investigate the potential mechanisms and therapies for bone formation in patients with infected bone defects.

Open bone fractures are generally contaminated by pathogenic microorganisms, including *Staphylococcus aureus*, *Staphylococcus epidermidis*, *streptococcus*, and *Klebsiella*, etc. [7]. Bacterial microorganisms will enter the fractured site and release coagulase or virulence factors, such as exotoxins and endotoxins, which may not only damage soft tissue or bone tissue but also hinder bone formation at the injury site by downregulating the osteogenic capacity of mesenchymal stem/stromal cells (MSCs) [8–10]. Recently, bone tissue engineering using MSCs has offered an alternative treatment approach for infected bone defect [11]. Bone regeneration in vivo is a complicated process, however, and demands a large number of MSCs in the fracture regions [12]. In addition, studies have indicated that composite scaffolds are superior to single-material scaffolds in terms of mechanical properties and bioactivity [13, 14]*.* Many researchers have thus attempted to modify single-material scaffolds with one or multiple materials for use in bone tissue engineering [13, 15, 16]. Compared with other widely used materials, porous hydroxyapatite (PHA) has gained interest because of its osteoinduction and osteoconduction properties in spite of its poor performance in cell adhesion and migration [14]. Another biomaterial of interest is alginate (ALG), a natural polysaccharide extracted from brown algae that is suggested to have antibacterial function [17]. In addition, fibronectin (FN) is also often used to modify the scaffold surface because of its cell adhesion properties [18]. In some studies, investigators have fabricated the PHA/ALG composite scaffold with the aim of possessing antibiotic function and good cell adhesion properties [19, 20]. Furthermore, when the composite scaffold PHA/ALG was modified to include FN (PHA/FN/ALG), it displayed fine properties for nerve regeneration [20]. In this study, we constructed a PHA/FN/ALG composite scaffold using layer-by-layer technology to possess antibiotic function and fine cell adhesive properties.

Studies have demonstrated that Wnt11 has been implicated in playing an important role in skeletal development [21]. Combined with transforming growth factor beta-1 (TGF-β1), Wnt11 may promote the chondrogenic differentiation of MSCs [22]. Furthermore, Wnt11 has been shown to promote osteoblast maturation and mineralization [23]. However, its role in osteogenesis of MSCs in an infectious environment remains to be elucidated.

We hypothesized that Wnt11 plays an important role in the osteogenesis of human mesenchymal stem/stromal cells (hMSCs) in an infected bone defect. Here, we fabricated a composite scaffold of PHA/ALG/FN. Using lentivirus technology, we also constructed hMSCs that overexpressed or silenced (via RNA interference) Wnt11. These modified hMSCs were then loaded on the PHA/ALG/FN composite scaffold and transplanted into an infected bone defect rabbit model. Subsequently, we monitored serological inflammatory markers using enzyme-linked immunosorbent assay (ELISA). One month after implantation, the osteogenesis capacity in different grafts was evaluated by X-ray photography, gross observation, micro-computed tomography (micro-CT), quantitative PCR, and histological analysis. We found that Wnt11 plays an important role in bone regeneration in an infectious environment.

## Methods

### Ethics statement

All experimental procedures were approved by the Kunming General Hospital Committee on Ethics for the care and use of laboratory animals. Before bone marrow collection, approval and informed consent were obtained from the Institutional Review Board and the donors.

### Culture of hMSCs

hMSCs were obtained according to a previously described method [24]. Briefly, approximately 8 ml bone marrow was aspirated from seven male and five female donors, 31–56 years old. Mononuclear cells were isolated by Percoll density gradient centrifugation (1.073 g/ml; Sigma, St. Louis, Missouri USA) at 900 × *g* for 20 minutes. The cells were rinsed with phosphate-buffered saline (PBS) and plated in a 25 cm^2^ cell culture flask (Costar Corning, NY, USA). The expansion medium used was Dulbecco’s modified Eagle’s medium/F12 (DMEM/F12; HyClone, Logan, Utah, USA) supplemented with 10 % fetal calf serum (HyClone) and 100 U/ml penicillin–streptomycin (HyClone). During culture, the medium was changed every 3 days. Cells were passaged after reaching 90 % confluence and then used for experiments (passage 1).

### Fabrication of PHA/FN/ALG composite scaffold

Cylindrical PHA (diameter: 0.5 cm; thickness: 1.0 cm) was provided by the Engineering Research Center in Biomaterials (Sichuan University, ChengDu, China). FN (10 μg/ml) and ALG (2 w/v %) solutions were prepared beforehand. PHA samples were initially immersed in FN solutions for 24 hours at 37 °C to obtain a precursor scaffold. The composite scaffold was formed by dipping PHA/FN into ALG solution for another 24 hours at 37 °C. The final composite scaffold of PHA/FN/ALG was prepared for subsequent assays after dehydration at 4 °C for 24 hours and radio-sterilization.

### Lentiviral vector construction and virus production

Lentiviral vector (pGC-LV-GFP) for expressing Wnt11 short hairpin RNA (Wnt11-shRNA-LV) was purchased from Genechem Corp. (Shanghai, China). The small interfering RNA (siRNA) sequence targeting Wnt11 was 5′-TGACTTCTGCATGAAGAAT-3′. As a control, we used a green fluorescent protein (GFP) lentiviral vector with a scrambled siRNA sequence (sequence: 5′-TACTGACGATGATACGTAT-3′). The designed oligonucleotide fragments were synthesized and cloned into pGC-LV-GFP vectors (GeneChem, Shanghai, China). The target recombinant lentiviral vector and empty vector were transfected and amplified in 293 cells using Lipofectamine 2000 (Invitrogen, Carlsbad, California, USA) according to the manufacturer’s instructions. Viral titers were collected from the supernatant of 293 cells and detected by optical density (OD). After being collected, the lentivirus was prepared at a titer of 10^9^ transfection units/ml for subsequent assays.

To obtain Wnt11 overexpression lentiviral vector, Wnt11 was synthesized and cloned into the pGC-LV-GFP vector (GeneChem). Target recombinant lentiviral vectors were also transfected in 293 cells and packaged into virus particles (LV-Wnt11). Viral supernatant was collected after 48 hours, and the Wnt11 gene expression level was determined by quantitative PCR.

### Transduction

hMSCs were seeded into six-well plates at a concentration of 5 × 10^5^ cells/ml in 2 ml medium for each well. These cells were then transfected with virus particles encoding Wnt11 RNAi or the Wnt 11 overexpression construct at a multiplicity of infection of 100 in complete culture medium supplemented with polybrene (5 μg/ml) using the Lipofectamine 2000 kit (Invitrogen) according to the manufacturer’s instructions. In addition, empty LV-GFP lentivirus was also transfected and served as a control. Seventy-two hours after transfection, the cells were collected for quantitative PCR or western blotting assay to determine the transfection efficacy. In addition, duplicate cells were also prepared for subsequent experiments.

### Western blot assay

Total protein was extracted from hMSCs using the Total Protein Extraction kit (Sigma) according to the manufacturer’s protocols. The protein concentration was determined with a BCA Protein Assay kit (Thermo scientific, Rockford, Illinois, USA) according to the manufacturer’s instructions. Then 40 μg protein were separated by SDS-PAGE, and then were transferred onto polyvinylidene difluoride membrane. The membrane was incubated with mouse polyclonal antibodies specific for Wnt 11 (1:150; Santa Cruz, CA, USA) and β-catenin (1:1000; Santa Cruz) overnight at 4 °C. After washing with Tris-buffered saline containing 0.1 % Tween, the membranes were incubated with secondary antibody (1:5000; Santa Cruz) for 1 hour. Finally, the membrane was exposed, visualized using an enhanced chemiluminescent kit (Merck Millipore, Eschborn, Germany) and a chemiluminescence detection system (Bio-Rad, Hercules,California, USA). Protein bands were quantified using the Quantity One software (Bio-Rad).

### Cell loading into the PHA/FN/ALG composite scaffold

After transduction, the hMSCs were trypsinized, rinsed, and resuspended with fresh medium. Cells (3 × 10^6^/ml) were loaded into the PHA/FN/ALG composite scaffold (0.5 cm diameter, 1.0 cm thick) and centrifuged at 50g for 1 minute. Cell-PHA/FN/ALG grafts were incubated at 5 % CO_2_ and 37 °C for 24 hours and then were induced for 3 days with osteogenic medium containing 100 nM dexamethasone, 0.2 mM ascorbate, and 10 mM β-glycerophosphate (Sigma). Finally, the cell-PHA/FN/ALG grafts were prepared for implantation.

### Cell adhesion assay

The cell adhesion ability was assessed as described previously [25]. Briefly, after labeling with 4′,6-diamidino-2-phenylindole (DAPI; Sigma) for 5 minutes, hMSCs were seeded onto the surface of the scaffold at a density of 10,000 cells/cm^2^. Subsequently, the scaffold was centrifuged at 20 × *g* for 5 minutes. After initial fluorescence readings were taken, the scaffold was inversely centrifuged at 20 × *g* for another 5 minutes. Post-spin fluorescence readings were used to determine the density of the adherent hMSCs.

### Cell proliferation assay

Evaluation of cell proliferation on the PHA/FN/ALG composite scaffold was performed as reported previously [18]. Briefly, cells were seeded onto the scaffold in a 24-well plate at a density of 3000 cells/cm and then were maintained in stem cell complete culture medium. Scaffold without cells was employed as a control. After culturing for 1, 3, 5, 7, and 9 days, 10 μl 3-(4, 5-Dimethylthiazol-2-yl)-2, 5-diphenyltetrazolium bromide (MTT) were added to every well. The cells were then incubated for 4 hours at 37 °C before 100 μl dimethyl sulfoxide solution was added. After mixing for another 10 minutes, the OD of every well was determined at 570 nm with the Thermo System (Varioskan Flash, Thermo, Massachusetts, USA). A total of three replicate wells were used to obtain the mean value. The final OD values were normalized to the control.

### *S. aureus* culture and animal model

The culture of *S. aureus* was performed following the common procedures described in our previous study [26]. Briefly, *S. aureus* was grown in tryptic soy broth at 37 °C with shaking and then centrifuged at 15,000 × *g* for 5  minutes, and finally resuspended in PBS. The bacterial number of the supernatant was determined the using tablet colony counting method before use in the experiments.

An animal model of osteomyelitis was created as described previously [27, 28]. Briefly, 2 weeks after *S. aureus* (2 × 10^6^ colony-forming units) was injected into the right tibia of rabbit, a drastic debridement was performed on the infectious tissues or sequestrums. Then, an infected bone defect on the tibia (0.7 cm wide, 1.0 cm long) was prepared for implantation surgery. In total, 35 New Zealand rabbits (of both sexes), each weighing 2.2–2.5 kg, were randomly divided into five groups, with seven animals in each group: group 1, blank control without graft implantation group (BCWG); group 2, only composite scaffold without cell implantation group (OSWG); group 3, empty lentivirus-transduced hMSCs/scaffold group (ELSG); group 4, Wnt11-RNAi hMSCs/scaffold group (WISG); and group 5, Wnt11 overexpressing hMSCs/scaffold group (WOSG). After implantation, all rabbits received intramuscular penicillin injections (400,000 units) once a day for 5 consecutive days. One month later, the rabbits were observed by standard X-ray imaging (Siemens, Berlin, Germany) and sacrificed for subsequent assays.

### Systemic immunological reactions

At different time points after implantation, a 5 ml blood sample was drawn from the auricular vein of each rabbit and the final serum was used to detect the systemic immune reaction. Immune cytokines, including interleukin (IL)-2, IL-4, IL-6, IL-10, interferon gamma (IFNγ), and TGF-β1, were measured using the ELISA Kit (R&D Systems, Minneapolis, MN, USA) according to the manufacturer’s instructions.

### Quantitative RT-PCR

Total RNA was extracted from transfected cells or scaffolds using Trizol reagent (Takara, Tokyo, Japan) according to the manufacturer’s protocols. After determining the RNA concentration, RNA (1 μg) was reverse transcribed into cDNA using a reverse transcription kit (Takara). Then 1 μl cDNA was amplified with a SYBR Green Kit (Takara) using the ABI 7500 Real-Time PCR Detection System (Applied Biosystems, Foster, California) in a 20 μl reaction system. The housekeeping gene GAPDH was employed as an internal control to normalize the reactions. Gene expression was determined by the 2^–ΔΔCt^ method. The primer sequences and reaction protocol are presented in Table 1.

**Table 1:** Primer sequences and procedure parameters used in the quantitative PCR analysis

Gene name	Primer sequence	Annealing temperature (Ta) (°C)	Cycles
β-actin	5′-GTGGGGCGCCCCAGGCACCA-3′ (forward)	56	42
	5′-CTTCCTTAATGTCACGCACGATTTC-3′ (reverse)		
Wnt11	5′-TGCAGGAGCTGCAGGATGTGG-3′ (forward)	59	39
	5′-AGCTCCATGGAGTGTCTCCAG-3′ (reverse)		
OC	5′-ATGAGAGCCCTCACACTCCTC-3′ (forward)	60	28
	5′-GCCGTAGAAGCGCCGATAGGC-3 (reverse)		
Runx-2	5′-ACGACAACCGCACCATGGT-3′ (forward)	60	28
	5′-CTGTAATCTGACTCTGTCCT-3′ (reverse)		
ALP	5′-TGGAGCTTCAGAAGCTCAACACCA-3′ (forward)	58	30
	5′-ATCTCGTTGTCTGAGTACCAGTCC-3′ (reverse)		
Osterix	5′-GCAGCTAGAAGGGAGTGGTG-3′ (forward)	58	42
	5′-GCAGGCAGGTGAACTTCTTC-3′ (reverse)		
Collagen I	5′-CCTGAGCCAGCAGATTGA-3′ (forward)	59	29
	5′-TCCGCTCTTCCAGTCAG-3′ (reverse)		
BSP	5′-AAGGCTACGATGGCTATGATGGT-3′ (forward)	61	30
	5′-AATGGTAGCCGGATGCAAAG-3′ (reverse)		

### Radiography and micro-CT analysis

To evaluate the graft position and the general bone formation in the tibia, X-ray imaging was performed 1 month after implantation. To assess the osteogenesis capacity of the grafts qualitatively and quantitatively, micro-CT was performed as described previously [29, 30]. The grafts were removed and evaluated using micro-CT (GE Company, London, Ontario, Canada) with the following parameters: 60 kV, 0.6 mm, 800 μA, and 150 players. More than 1000 slice images were obtained and were reconstructed at the spatial nominal resolution of 10 μm. The newly formed bone was separated from the grafts by setting the threshold at 750–1200 HU. The grafts were evaluated for osteogenesis capacity based on the following morphometric indices: bone mineral content (BMC), bone mineral density (BMD), tissue mineral density (TMD), tissue mineral content (TMC), bone volume fraction (BVF), and bone volume (BV).

### Gross observation and histological analysis

Rabbits were sacrificed with an overdose of sodium pentobarbital 1 month after implantation. The specimen was harvested for gross observation and then immediately fixed in 10 % neutral buffered formalin for 24 hours. After sequential dehydration in ethanol solutions, the specimen was embedded in PMMA solution for 1 week and then sectioned to 50 μm thick by a diamond saw (Leica-LA, Heidelberg, Baden-Wurttemberg, Germany). The slices were stained with Villanueva-Goldner’s trichrome (VG) and observed in a blind fashion. In every group, three graft-derived slices were randomly chosen to quantitatively analyze the newly formed bone and collagen I using Image-Pro Plus (Silver Spring, Maryland, USA) software 6.0. Osteogenesis capacity was quantified in terms of the area of light blue and red, which represented collagen I and newly formed trabecular bone, respectively [31].

### Statistical analysis

Data are presented as the mean ± standard deviation (SD). Statistical analysis for differences between groups was performed using one-way analysis of variance. In addition, an independent-samples *t* test was used to compare any two groups. *p* <0.05 was considered statistically significant.

## Results

### hMSC culture, transfection and transfection efficiency

**Fig. 1:**
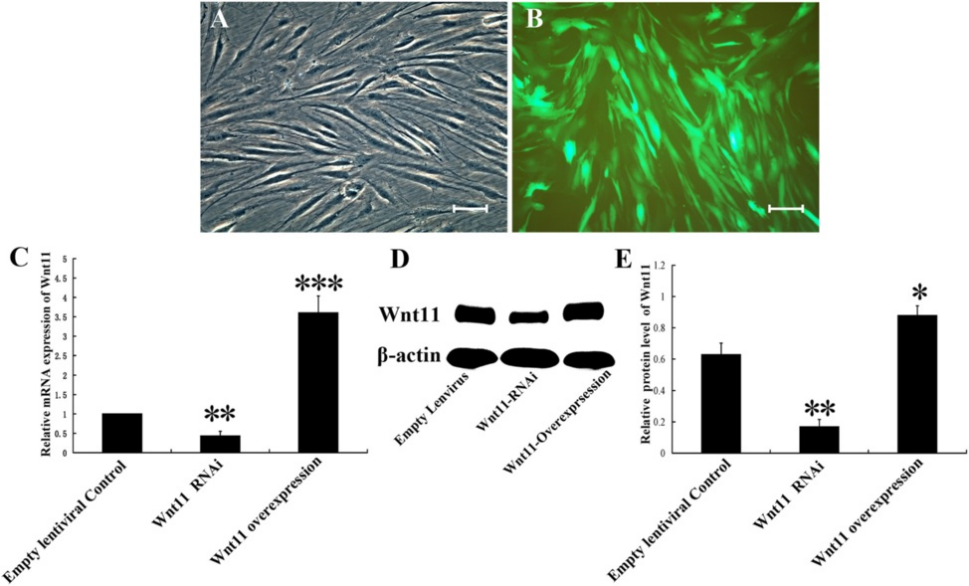
hMSCs were cultured in vitro and infected with lentiviral vectors targeting Wnt11. **a** Passage 0 hMSCs show the typical shape of MSCs. **b** Representative images of hMSCs with lentivirus transduction. **c** Quantitative PCR assay of the Wnt11 gene expression level in Wnt11 RNAi-expressing or Wnt1-overexpressing hMSCs after transduction; an empty vector expressing enhanced GFP served as a control. **d** Western blot assay of the Wnt11 protein expression of Wnt11 RNAi-expressing or Wnt1-overexpressing hMSCs after transduction; an empty vector expressing enhanced GFP served as a control. **e** Quantitative analysis of the relative protein level of Wnt11; results were based on the density and normalized to β-actin. Data presented as mean ± SD. *p <0.05, **p <0.01 and ***p <0.001 compared with empty vector control. Scale bar = 50 μm

hMSCs (Fig. 1a) were cultured and passaged when they reached 90 % confluence. Passage 1 cells were transfected with pGC-LV-GFP vectors designed to overexpress or silence Wnt11 (Fig. 1b). After transfection, all of the cells, which received different treatments, were used to determine transfection efficiency by quantitative PCR and western blot assay (Fig. 1c–e). Quantitative PCR indicated that lentivirus-mediated Wnt11 RNAi in hMSCs significantly diminished the Wnt11 gene expression level in comparison with the control, whereas the hMSCs designed to overexpress Wnt11 showed significantly higher expression levels than the control cells (empty lentivirus transduction): the suppression or stimulation rate was 57 % and 260 % after transfection, respectively (Fig. 1c). Significant differences in Wnt11 expression were found between the RNAi group and the overexpression and control groups (Fig. 1c). Furthermore, western blot analysis also revealed significant downregulation of Wnt11 protein levels in the RNAi group and upregulation in the overexpression group when compared with the control group (Fig. 1d, e).

The results suggest that lentivirus-mediated Wnt11 RNAi may successfully inhibit Wnt11 expression. Furthermore, the lentivirus-mediated Wnt11 overexpression construct could increase the Wnt11 level effectively both at the mRNA and protein levels.

### Cell adhesion assay and cell proliferation assay

**Fig. 2:**
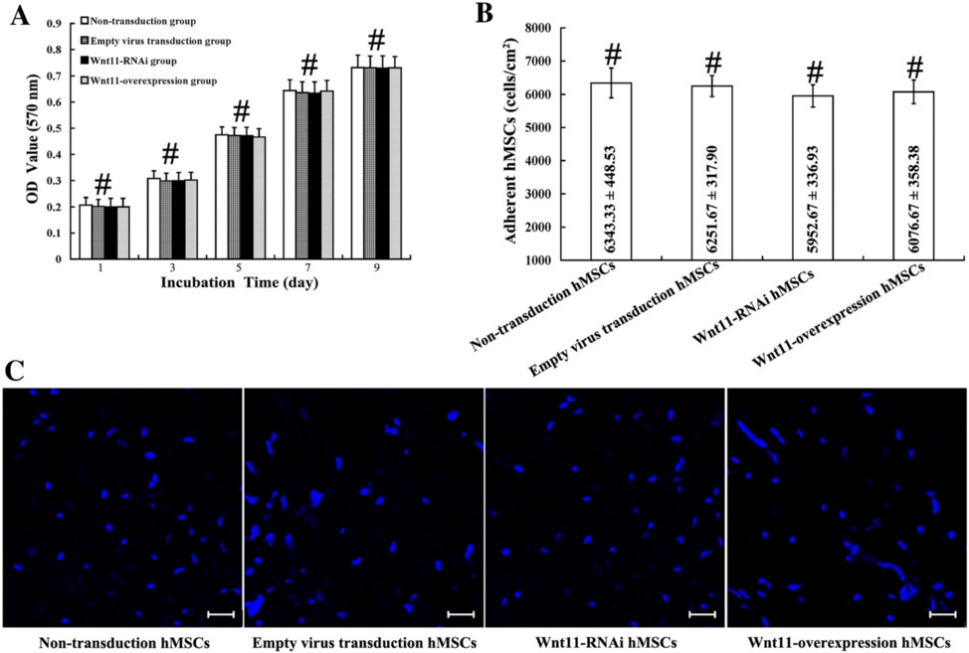
Proliferation and adhesion assay of hMSCs on the surface of PHA/FN/ALG composite scaffolds. **a** hMSC proliferation assay. **b** hMSC adhesion assay. **c** Distribution of DAPI-labeled hMSCs adhered to the scaffolds. Data presented as mean ± SD. ^#^ *p* >0.05. Scale bar = 100 μm. *hMSC* human mesenchymal stem/stromal cell, *OD* optical density

The OD value reflects metabolically active cells. As shown in Fig. 2a, hMSCs loading on the composite scaffolds showed a gradual increase in metabolically activity over time. At any time point, different hMSCs displayed similar metabolically active, and no significant differences among them could be found (*p* >0.05) (Fig. 2a).

The strength of cell adhesion was determined by measuring the density of fluorescently labeled cells after centrifugation. The adhesive cell number was calculated and standardized by the fluorescent difference between the initial readings and the second readings. The results showed that the composite scaffolds exhibited similar capacity to bind to different hMSCs; no significant differences could be observed (*p* >0.05) (Fig. 2b, c).

### Cell loading in scaffolds and implantation

**Fig. 3:**
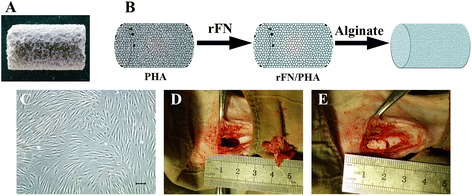
PHA was modified with FN and ALG and was transplanted into the infected bone defect after loading with Wnt11-modified MSCs. **a** Gross appearance of PHA. **b** Schematic illustration of PHA modified with FN and ALG. **c** Representative shape of the transfected MSCs. **d** An infected bone defect (1.0 cm long and 0.5 cm in width) was made for implantation. **e** PHA/FN/ALG with or without Wnt11-modified MSCs were transplanted into the bone defect site. Scale bar = 50 μm. *rFN* recombinant fibronectin, *PHA* porous hydroxyapatite

As shown in the schematic diagram, PHA displayed a porous and crude construction, but after sequential surface decoration with ALG and FN, PHA formed a comparatively smooth surface (Fig. 3a, b). Those transfected hMSCs still showed a spindle-like shape and then were seeded on the scaffolds as seed cells (Fig. 3c). Two weeks after *S. aureus* injection, the tibia became infectious before implantation (Fig. 3d). After extensive debridement, infectious tissues and sequestrums were cleared out to prepare the graft bed (1.0 cm × 0.6 cm) (Fig. 3d, e). Those scaffolds were implanted into the bone graft bed for subsequent evaluation (Fig. 3d, e).

### Systemic immunological reactions

**Fig. 4:**
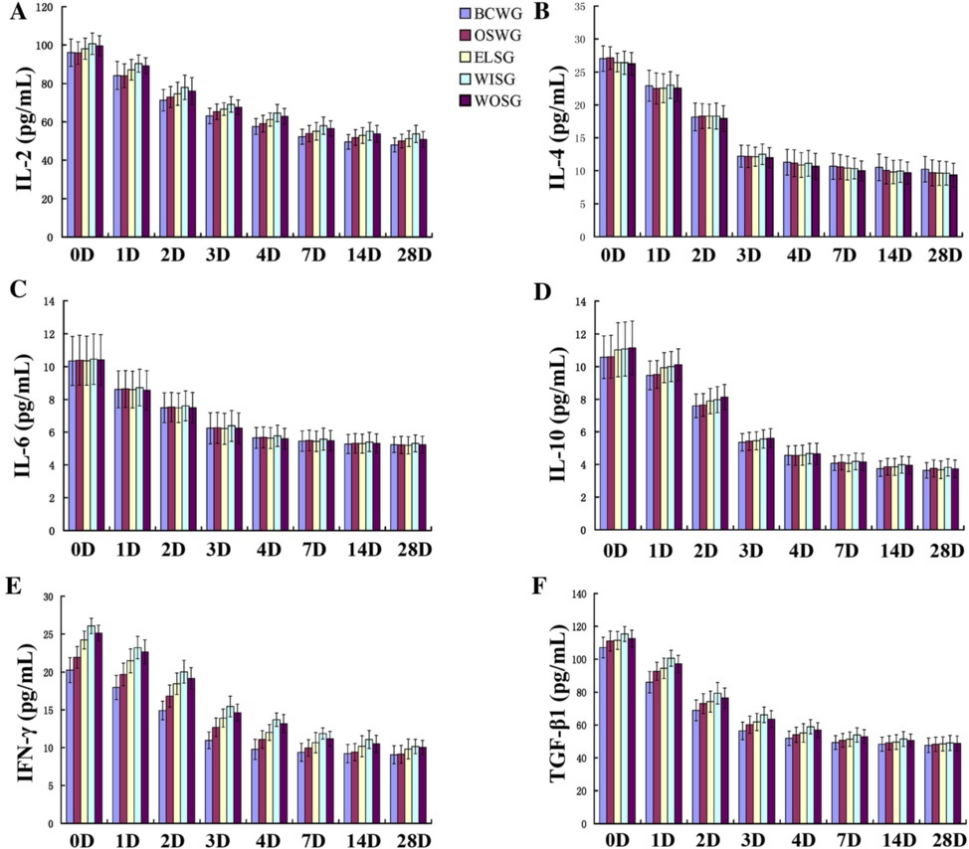
Systemic immunological reactions were detected postoperatively, and the serum concentrations of IL-2 **a**, IL-4 **b**, IL-6 **c**, IL-10 **d**, IFNγ **e**, and TGF-β1 **f** were measured at 0, 1, 2, 3, 4, 7, and 14, 28 days after transplantation. No significant differences existed between any two groups at the same time points (*p* >0.05, *n* = 3). Data presented as mean ± SD. *BCWG* blank control without grafts implantation group, *ELSG* empty lentiviral vectors transduction hMSCs/scaffold group, *IFNγ* interferon gamma, *IL* interleukin, *OSWG* only scaffold without cells implantation group, *TGF-β1* transforming growth factor beta-1, *WISG* Wnt11 RNAi hMSCs/scaffold group, *WOSG* Wnt11-overexpressing hMSCs/scaffold group

Three rabbits expired during the experiments: one died pre implantation and two died post implantation. After implantation, the ELISA results of serum samples indicated a gradual decline in the IL-2, IL-4, IL-6, IL-10, IFNγ, and TGF-β1 levels (Fig. 4a–f). Interestingly, these inflammatory cytokines declined more significantly in the first 3 days post implantation than in the subsequent days (Fig. 4a–f).

### Evaluation of osteogenesis by quantitative RT-PCR

**Fig. 5:**
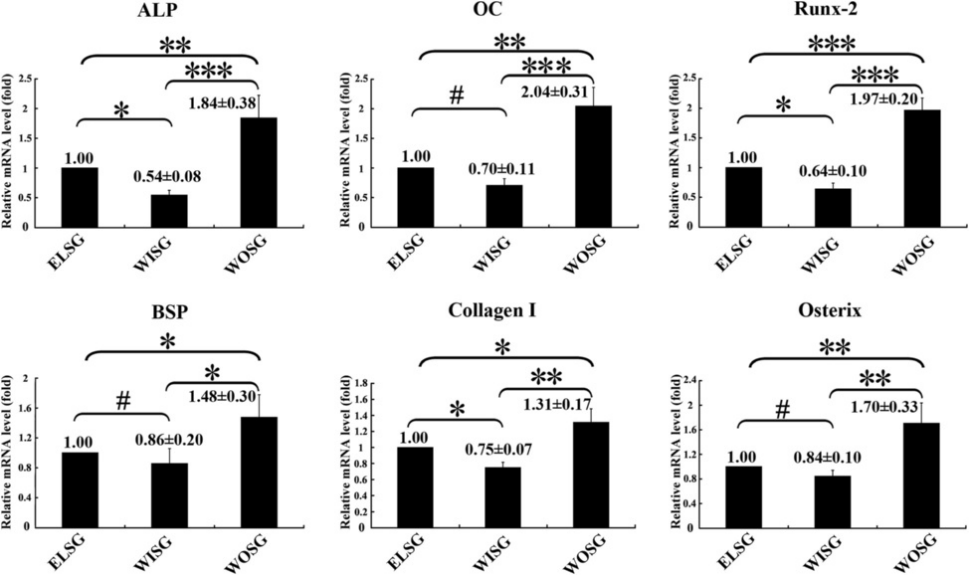
Expression of relative osteogenic genes in grafts 1 month post operation were tested by quantitative PCR. Data presented as mean ± SD. *p <0.05, **p <0.01, ***p <0.001, ^#^ *p* >0.05; *n* = 3. *ELSG* empty lentiviral vectors hMSCs/scaffold group, *WISG* Wnt11 RNAi hMSCs/scaffold group, *WOSG* Wnt11-overexpressing hMSCs/scaffold group

To investigate osteogenesis in the infected bone defect at gene level, quantitative RT-PCR was performed at 1 month post implantation. As shown in Fig. 5, all six osteogenic-related genes (ALP, OC, Runx-2, BSP, Collagen I, and Osterix) displayed a similar expressional trend among different groups. Nearly every gene displayed high levels in WOSG, low levels in WISG, and an intermediate level in ELSG. Furthermore, except for changes in OC, BSP, or Osterix between ELSG and WISG, significant differences were observed between any other two groups for each gene (*p* <0.05).

### Radiography

**Fig. 6:**
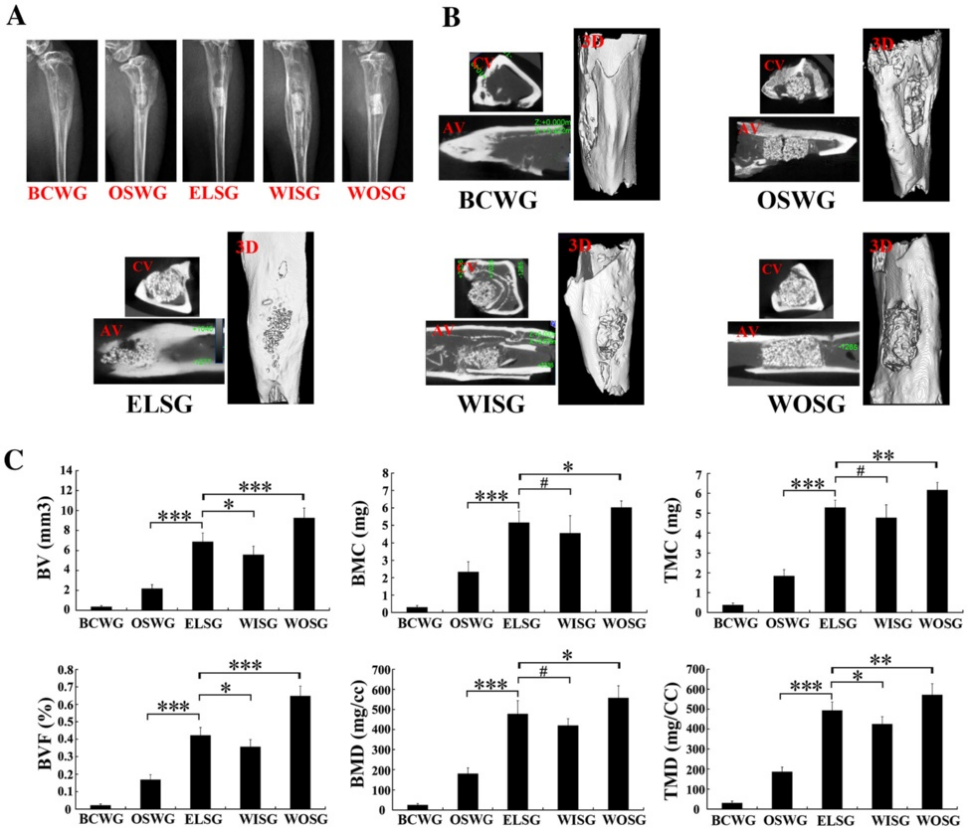
Radiographic observation and micro-CT analysis on the osteogenesis of grafts 1 month post implantation. **a** X-ray observation. **b** Axial view scanning, coronal view scanning, and three-dimensional reconstruction by micro-CT 1 month post implantation. **c** Quantitative analysis of osteogenesis in grafts. Data presented as mean ± SD. *p <0.05, **p <0.01, ***p <0.001, ****p <0.0001, ^#^ *p* >0.05; *n* = 3. *AV* axial view, *BCWG* blank control without grafts implantation group, *BMC* bone mineral content, *BMD* bone mineral density, *BV* bone volume, *BVF* bone volume fraction, *CV* coronal view, *3D* three dimensional, *ELSG* empty lentiviral vectors transduction hMSCs/scaffold group, *OSWG* only scaffold without cells implantation group, *TMC* tissue mineral content, *TMD* tissue mineral density, *WISG* Wnt11 RNAi hMSCs/scaffold group, *WOSG* Wnt11-overexpressing hMSCs/scaffold group

Radiographic standard X-ray imaging indicated that the radio-opaque area or the radiopacity at the original bone defect sites could be visualized clearly (Fig. 6a). The radiographic findings revealed weak infection signs, including small sequestration and periosteal reaction around the grafts or the defect places (Fig. 6a). However, it was interesting to note that the radio-translucent area at the original defect bed decreased gradually in the group order BCWG > OSWG > WISG > ELSG > WOSG (Fig. 6a).

To further analyze the osteogenic capacity, micro-CT was carried out to determine the quality and quantity of osteogenesis capacity (Fig. 6b, c). From the axial view, coronal view, and three-dimensional view, the results demonstrated that the bone defects had been repaired structurally to different degrees (Fig. 6b, c). For the quantity of regenerated bone in the scaffold, the data indicated that all six indexes of osteogenesis in ELSG were significantly higher than in both BCWG and OSWG (*p* >0.05, Fig. 6b, c). The results quantitatively further revealed that all six indexes in WOSG were significantly higher than those in both ELSG and WISG (*p* <0.05, Fig. 6b, c). Moreover, the osteogenesis indexes in ELSG were also higher than those in WISG, and significant differences could be observed in the indexes of BV, BVF, and TMD (*p* <0.05, Fig. 6b, c), but did not in the BMC, BMD, and TMC indexes (*p* >0.05, Fig. 6b, c).

### Gross observation

**Fig. 7:**
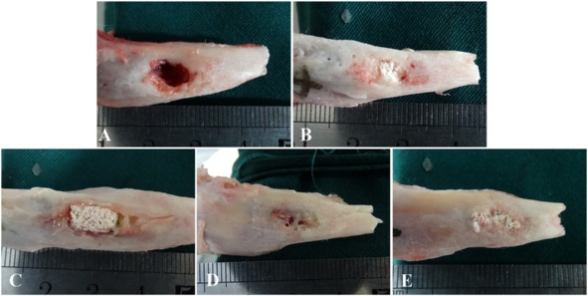
Gross observation of those grafts 1 month after transplantation. **a** Blank control without grafts implantation group. **b** Only scaffold without cells implantation group. **c** Empty lentiviral vectors transduction hMSCs/scaffold group. **d** Wnt11 RNAi hMSCs/scaffold group. **e** Wnt11-overexpressing hMSCs/scaffold group

Gross morphology was observed at 1 month after implantation (Fig. 7). In BCWG, no newly formed bone was observed in the original lacuna of the bone defect, with the exception of a very small amount of inflammatory fibrous tissue (Fig. 7a). As for OSWG, fractures could be observed on the surface of the scaffold and no significant bone-like hard tissue formed, with the exception of a large amount of inflammatory granulation tissue (Fig. 7b). In ELSG, the scaffold was intact and surrounded with a moderate amount of inflammatory granulation tissue (Fig. 7c). However, the scaffold in WISG was covered by more inflammatory granulation and purulent tissue; moreover, the surface of the defect was vague between the boundary between the scaffold and the tibia (Fig. 7d). Interestingly, the defect region in WOSG was filled with the intact scaffold, which was attached with fibrous tissue, and the boundary between the scaffold and the tibia completely vanished (Fig. 7e).

### Histological assessment

**Fig. 8:**
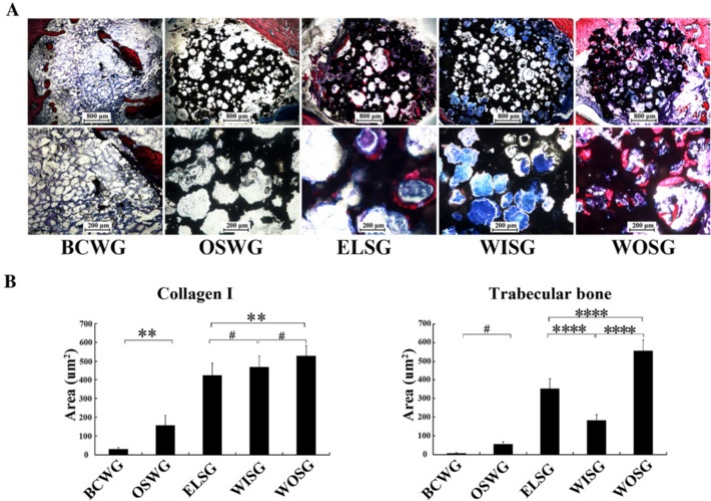
Histological evaluation of grafts 1 month after implantation. **a** VG staining; collagen I and trabecular bone shown in *blue* and *red*, respectively. **b** Quantitative analysis based on the area of new bone tissue. Data presented as mean ± SD. **p <0.01, ***p <0.001, ****p <0.0001, ^#^ *p* >0.05. *BCWG* blank control without graft implantation group, *ELSG* Empty lentiviral vectors hMSCs/scaffold group, *OSWG* only scaffold without cell implantation group, *WISG* Wnt11 RNAi hMSCs/scaffold group, *WOSG* Wnt11-overexpressing hMSCs/scaffold group

To analyze the osteogenesis capacity, VG stain was employed to microscopically detect hydroxyapatite, collagen I and newly formed trabecular bone, which were dyed black, blue or purple and red, respectively (Fig. 8a). The BCWG grafts demonstrated almost no collagen I or newly formed trabecular bone in the defect bed (Fig. 8a). For OSWG, besides very little collagen I, no trabecular bone could be observed in the macropores of the scaffolds (Fig. 8a). As for the other three groups, when compared with WISG, ELSG showed significantly more trabecular bone but less collagen I appeared in the scaffold (Fig. 8a, b). However, more collagen I and newly formed bone were observed in WOSG than in both ELSG and WISG (*p* <0.0001, Fig. 8a, b).

## Discussion

In clinical practice, quite a few open fractures generally develop into infected nonunion [32]. Studies have confirmed that *S. aureus* is the main pathogenic bacterium which causes infected bone nonunion or osteomyelitis [8]. During debridement, nearly all dead bone or tissue needs to be eliminated, which inevitably leads to infected bone defect [33]. In this study, rabbits were injected in the tibia with *S. aureus* to induce the pathogenesis of osteomyelitis disease [34]. In the early stage after implantation, serum inflammatory markers indicated significant highly systemic inflammation reaction in the infected rabbits. The results from X-ray imaging, gross observation, systemic immunological factors, and subsequent histological evaluation verified that the rabbits exhibited characteristics of infected bone defect. Those results suggested that an infected bone defect model was reliably made and could be used to evaluate bone formation of tissue-engineered bone.

The defect size of the bone defect model often ranged from 0.5 to 2.0 cm. In the present study, rabbits were treated by radical debridement to provide a bone defect 1.0 cm long. In addition, one study found that PHA without cells displayed no obvious bone repair function in vivo [11]. In the blank control group of this study, the results of micro-CT and histological analysis also supported this finding, whereas other hMSC-containing groups showed fine bone repair efficiency comparatively. Hence, in this study, the implanted tissue-engineered bone played an essential role in the bone repair process.

Current scaffold materials are not perfect for treating infected bone defect [14]. To obtain good scaffolds, researchers often modify existing materials by adding metal components such as zinc or titanium or other materials to endow the original scaffold with potent antibiotic efficiency [25, 35]. However, modified scaffolds could produce potential cytotoxicity or biocompatibility problems [36]. Hence, developing optimal scaffolds for treating infected bone defect is necessary before clinical use [37]. PHA has often been adopted for bone tissue engineering research or practical application due to its biocompatibility and bone induction and bone conduction properties [38]. However, when it was used for infected bone defect, PHA might have a reduced efficacy because it does not have antibiotic activity [39]. Furthermore, when compared with collagen-coated PHA, pure PHA is not easy for cells to adhere on, which might be a drawback for use as a scaffold [40, 41]. Moreover, both FN and ALG have short degradation periods and could be absorbed easily by tissues within 1 month in vivo [40, 42]. Hence, in this study we created a modified PHA with FN and ALG. Because the degradation period of PHA is longer than 4 months, PHA may still provide a support role as a scaffold in vivo within the 1-month observation time, even though FN and ALG have already degraded during that timeframe [43]. In general, our studies showed no significant drawbacks regarding the biological properties of the PHA/FN/ALG composite scaffold. However, our study did not completely eradicate the infected bone defect or completely repair the bone. Thus, better scaffold materials with potent osteogenesis capacity require further investigation. Furthermore, exploring the optimization of scaffolds with better cell–scaffold interfaces for treating infected bone defect is another important area of future research.

hMSCs are generally applied in bone tissue engineering for its multilineage differentiation capacities, and also display encouraging results [44, 45]. However, gene-modified hMSCs might be better for bone tissue engineering applications even in infectious microenvironments [46]. In this study, using gene silencing (RNAi) and gene overexpression methods based on lentivirus mediation, we successfully illuminated the role of the Wnt11 gene during the osteogenesis of hMSCs in an infectious environment in vivo. After 3 days of transduction, the results of quantitative PCR indicated that the Wnt11-RNAi hMSCs showed significantly lower Wnt11 gene level than that of the empty lentivirus-transduced hMSCs, and Wnt11-overexpressing hMSCs showed significantly higher expression than the empty-lentivirus hMSCs at the mRNA level. Additionally, these results were consistent with the protein expression analysis. Hence, these results indicated that Wnt11 expression was successfully modified. Furthermore, to shed light on the role of the Wnt11 gene in hMSCs, we constructed tissue-engineered bone with PHA/FN/ALG scaffolds containing different modified hMSCs in vitro. We then transplanted these scaffolds into an infected bone defect model. One month post implantation, the six examined osteogenic-related genes displayed similar trends across groups. The gene levels were significantly higher in WOSG but lower in WISG, when compared with ELSG. Based on the osteogenic differences among those different groups, it might be deducted that the Wnt11 gene plays an important role in hMSCs during osteogenic differentiation in an infectious environment.

Micro-CT is widely employed for the quantitative and qualitative evaluation of newly formed bone without physical disruption of the sample in vivo [47]. The parameters BV, BVF, BMC, BMD, TMC, and TMD reflect the bone quality indirectly [48]. Here, micro-CT results also revealed that all those parameters were significantly higher in WOSG than in any other groups. However, in ELSG the results showed inferior superior osteogenesis capacity compared with WISG. Hence, the results further indicated that Wnt11 overexpression enhanced the osteogenesis capacity of hMSCs, but Wnt11 RNAi attenuated the capacity of hMSCs in the infected bone defect model. Furthermore, histological analysis revealed that newly formed collagen I and trabecular bone in WOSG grafts were significantly higher than in the ELSG or WISG grafts. These results further suggested that Wnt11 overexpression may enhance the osteogenesis of hMSCs in the infectious environment.

As a transcription factor, TGF-β1 is involved in immune inflammatory reactions, proliferation or differentiation regulation, as well as promoting osteoblasts or inhibiting osteoclast in proliferation in a concentration-dependent manner [49, 50]. Here, our results showed that these inflammatory factors were downregulated 3 days after implantation, which indicates that a serious systematic inflammatory reaction occurred. The results demonstrated that the infected bone defect model was successful and an infectious microenvironment was created.

In spite of the merits and encouraging results, however, there are still some limitations. First, although WOSG displayed the strongest osteogenic potential, an obvious large massive bone to bridge the defect region and the original bone was not observed. Second, the in vivo inflammatory environment of rabbits is not completely similar to that of humans; thus, the results cannot be completely translated to humans. Third, the “golden standard” autologous bone graft was not performed in our model because this is a preliminary study on infected bone defect and our aim is to investigate the role of the Wnt11 gene in hMSCs. In our future studies, based on the current foundation, autologous bone grafting combined with larger animal models will be investigated.

## Conclusions

To our knowledge, this is the first study to investigate the role of Wnt11 during bone formation in an infectious environment. Our results demonstrated that Wnt11 plays an important role and may enhance the osteogenesis capacity of hMSCs in an infected bone defect model. Our findings may contribute to illustrating the mechanism of osteogenesis in an infectious environment and might provide new mechanisms for treating infected bone defect.

## Abbreviations

ALG: Alginate
BMC: Bone mineral content
BMD: Bone mineral density
BV: Bone volume
BVF: Bone volume fraction
CT: Computed tomography
DAPI: 4′,6-Diamidino-2-phenylindole
DMEM: Dulbecco’s modified Eagle’s medium
ELISA: Enzyme-linked immunosorbent assay
FN: Fibronectin
GFP: Green fluorescent protein
hMSC: Human mesenchymal stem/stromal cell
IFNγ: Interferon gamma
IL: Interleukin
MSC: Mesenchymal stem/stromal cell
OD: Optical density
PBS: Phosphate-buffered saline
PHA: Porous hydroxyapatite
PMMA: Polymethyl methacrylate
SD: Standard deviation
siRNA: small interfering RNA
TGF-β1: Transforming growth factor beta-1
TMC: Tissue mineral content
TMD: Tissue mineral density
VG: Villanueva-Goldner’s trichrome
